# Gentamicin removal in submerged fermentation using the novel fungal strain *Aspergillus terreus* FZC3

**DOI:** 10.1038/srep35856

**Published:** 2016-10-24

**Authors:** Yuanwang Liu, Huiqing Chang, Zhaojun Li, Cheng Zhang, Yao Feng, Dengmiao Cheng

**Affiliations:** 1 Institute of Agricultural Resources and Regional Planning, Chinese Academy of Agricultural Sciences, Key Laboratory of Plant Nutrition and Fertilizer, Ministry of Agriculture, Beijing, 100081, China; 2 Henan University of Science and Technology, Luoyang, 471003, China

## Abstract

Social concern and awareness of the potential risk posed by environmental residues of antibiotics such as gentamicin in the development of antibiotic resistance genes have increased. The present study used laboratory-scale experiments to develop methods for gentamicin removal from the environment. A fungus, strain FZC3, which could remove gentamicin in submerged fermentation, was isolated from solid waste and sewage water from a gentamicin production factory. The fungus was identified as *Aspergillus terreus* by sequencing the PCR-amplified ITS fragments of its rRNA-coding genes and by its morphology. The gentamicin removal efficiency exceeded 95% by day 7 under optimized culture conditions. The results showed that both biosorption and biodegradation were involved. We speculated that *Aspergillus terreus* FZC3 absorbed gentamicin and subsequently degraded it. We also found that *Aspergillus terreus* FZC3 survived and maintained a high bioremediation efficiency over a wide pH range, indicating its potential for future use in the large-scale bioremediation of gentamicin.

Gentamicin is an aminoglycoside antibiotic that is widely used, especially in China, to prevent human disease and promote livestock growth^1^. Millions of tons of waste water and solid waste containing gentamicin are annually generated during gentamicin production and in animal husbandry, and this can induce the development of gentamicin resistance genes in the environment^2^,^3^. Therefore, the development of methods for gentamicin removal from solid waste and waste water residues is critically important.

Gentamicin is heat stable and resistant to both acidic and alkaline conditions, which provides a challenge for gentamicin removal from the environment using common chemical degradation or physical methods^4^. In contrast, bioremediation provides the best and most economically feasible solution for gentamicin removal from waste. Antibiotics usually specifically inhibit bacteria, however, most fungi can survive in substrates containing antibiotics, and fungi have been widely used to remove organic pollutants including antibiotics via biosorption and biodegradation^5^,^6^,^7^,^8^,^9^,^10^,^11^,^12^,^13^,^14^. Fungi that can efficiently degrade gentamicin may exist in waste produced during the factory production of gentamicin. The main objective of the present study was to isolate, screen, and identify effective gentamicin-eliminating fungi from gentamicin production waste. The ability of fungi to remove antibiotics is affected by various environmental factors such as pH and temperature^15^, so an additional objective was to determine the optimal environmental conditions for the fungus to remove gentamicin from various substrates. A final objective was to determine the mode of action of bioremediation because this may affect the toxicity of the degradation products and the subsequent disposal methods for the fungus-antibiotic products.

## Results and Discussion

### Isolation and screening of gentamicin-removing fungi

**Figure 1:**
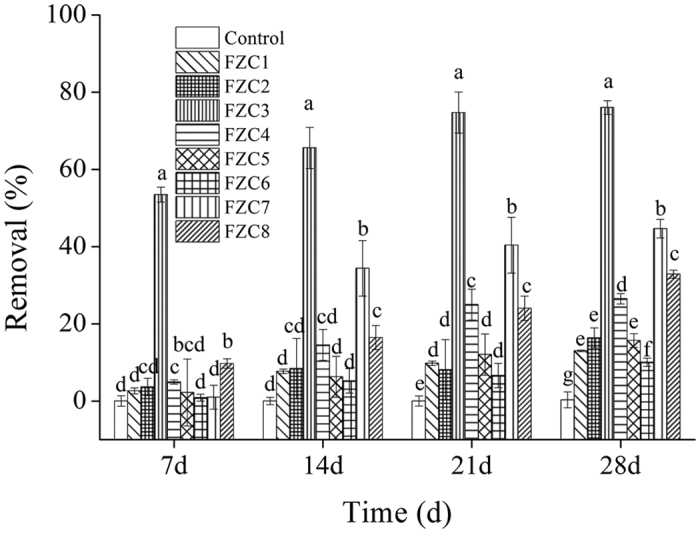
Gentamicin removal by FZC3 in 1/10 LPD with 100 mg L^−1^ gentamicin incubated at 30 °C and 150 rpm orbital shaking. The mean values and standard deviations (*error bars*) for seven day intervals are presented (n = 3). The figure was created by Origin 8.5.

Eight fungal strains were isolated from gentamicin production wastes. These fungi did not grow in liquid mineral salt medium (MSM) or remove any gentamicin in that medium, indicating that none of them could use gentamicin as a sole carbon source. All these fungi grew in a 10-fold dilution of liquid potato dextrose medium (LPD) and had gentamicin removal rates ranging from 10.1–76.1% (Fig. 1). The highest removal rate was found for fungal strain FZC3 (76.1%), suggesting that FZC3 removed gentamicin by co-metabolism in submerged fermentation. Strain FZC3 was therefore used in all subsequent experiments. Most (53.5%) of the gentamicin was removed during the first 7 days. An additional 12% and 8% of gentamicin were removed between 8 to 14 days and 15 to 21 days, respectively. Only 2.6% of gentamicin was removed between 22 to 28 days. The decline in gentamicin removal after longer incubation times in batch culture was likely due to the nutrient limitation and accumulation of waste products.

### Taxonomic identification of FZC3

**Figure 2:**
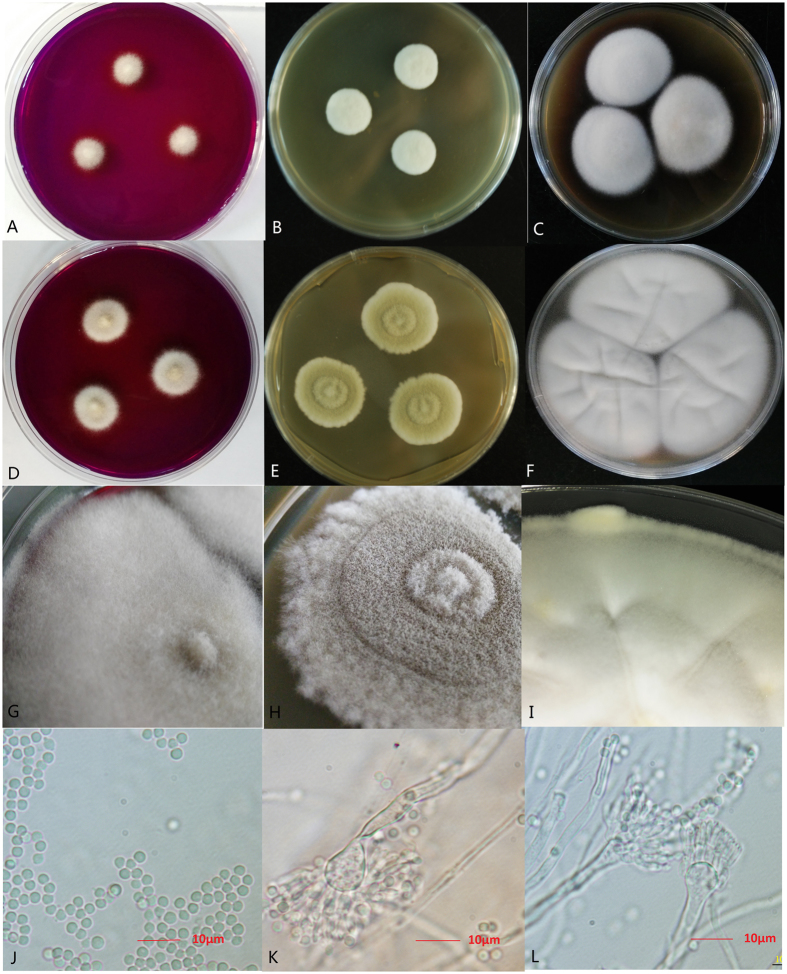
Morphological observation of *Aspergillus terreus* FZC3. (**A–C**): Colonies of *Aspergillus terreus* FZC3 incubated at 25 °C for 7d (**A**), CREA; (**B**), MEA; (**C**), CYA). (**D–F**): Colonies incubated at 37 °C for 7d (**D**), CREA; E, MEA; F, CYA). (**G–I**): Colonies incubated at 37 °C for 14 d (**G**), CREA; H, MEA; I, CYA). (**J–L**): Conidia and conidiophores as observed through a microscope (model: OLYMPUS B × 51). The figure was created by Adobe Photoshop CC.

The gene sequence of FZC3 was compared to other fungal sequences in the National Center for Biotechnical Information (NCBI, http://www.ncbi.nlm.nih.gov) database. The sequence alignment results showed that FZC3 had a 99% similarity with *Aspergillus terreus*. The morphological characteristics of the colonies and cells of FZC3 are shown in Fig. 2. Fluffy white colonies 0.8–1.2 cm and 1.6–2.0 cm in diameter grew at 25 °C and 37 °C, respectively, on creatine agar (CREA) plates in 7 days. FZC3 colonies on malt extract agar (MEA) plates were white and 1.6–2.8 cm in diameter at 25 °C and were 2.6–2.9 cm with brown stratification at 37 °C by the 7th day. On Czapek yeast autolysate (CYA), FZC3 fan-shaped colonies with radii of 2.8–3.1 cm and 4.0–4.2 cm grew at 25 and 37 °C, respectively. Micrographs of an MEA culture showed globose, hyaline conidia of 2.5–3.0 μm in diameter on day 7 of incubation. The globose vesicles were 6.5–9.0 μm in diameter and had a stipe width of 2.5–4.5 μm. These characteristics were in good agreement with the morphology of *Aspergillus terreus* as described by Samson^16^. Based on its morphological characteristics and gene sequence, FZC3 was classified as *Aspergillus terreus*.

### Parameter optimization

**Figure 3:**
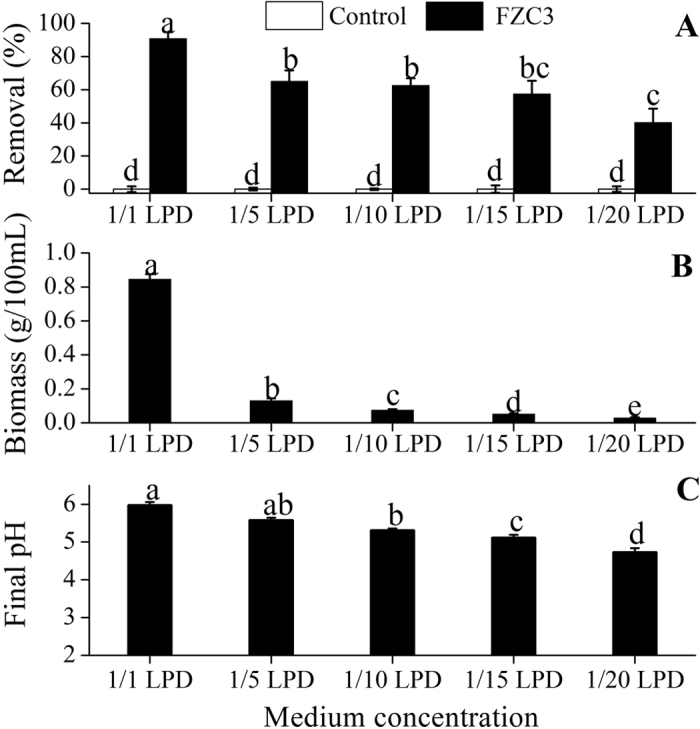
Effects of the medium concentration on the gentamicin removal, the biomass of FZC3 and the final pH of the medium. (**A**) Gentamicin removal by day 7 in response to different medium concentrations. (**B**) Biomass of FZC3 in 100 mL of medium by day 7 in response to different medium concentrations. (**C**) Final pH of the medium by day 7 in response to different medium concentrations. The mean values and standard deviations (*error bars*) are presented (n = 3). *Data bars* with the same letter are not significantly different from one another, as determined by Duncan’s test (*p* < 0.05). The figure was created by Origin 8.5.

In order to improve the ability of *Aspergillus terreus* FZC3 to remove gentamicin and develop its potential for use in large-scale applications, the fermentation parameters, including the LPD medium concentration, the gentamicin concentration, the shaking frequency, the inoculum size, the temperature and the initial pH were optimized in the laboratory. The LPD medium concentration had a large impact on gentamicin removal by FZC3. The gentamicin removal rate decreased from 91% to 40% when the medium was diluted from 1/1 to 1/20 (Fig. 3A). The biomass of *Aspergillus terreus* FZC3 in 100 mL of medium was also significantly reduced from 0.84 g to 0.03 g (Fig. 3B). The final pH decreased from 6 to 4.7 (Fig. 3C). It has been previously demonstrated that *Aspergillus terreus* could effectively remove metals and dyes via sorption and degradation and that the process was highly related to the fungal biomass produced^10^,^17^. Similarly, in our study, the medium concentration likely affected gentamicin removal via effects on fungal biomass production - a higher LPD concentration produced greater fungal biomass, resulting in a higher gentamicin removal rate. More biomass produces a larger adsorption surface and more catabolic enzymes. As the medium was diluted with deionized water, its buffering capacity might be weakened compared with the original medium. Active fungi growth usually lowers the pH^18^ and that had a greater impact in dilute than in undiluted medium, so the low gentamicin removal rate in dilute medium may be partly due to the lower pH. Gentamicin removal also decreased the pH. Because a lower pH may increase the difficulty for industrial bioremediation application, adjusting the pH of the reaction solution will be essential.

**Figure 4:**
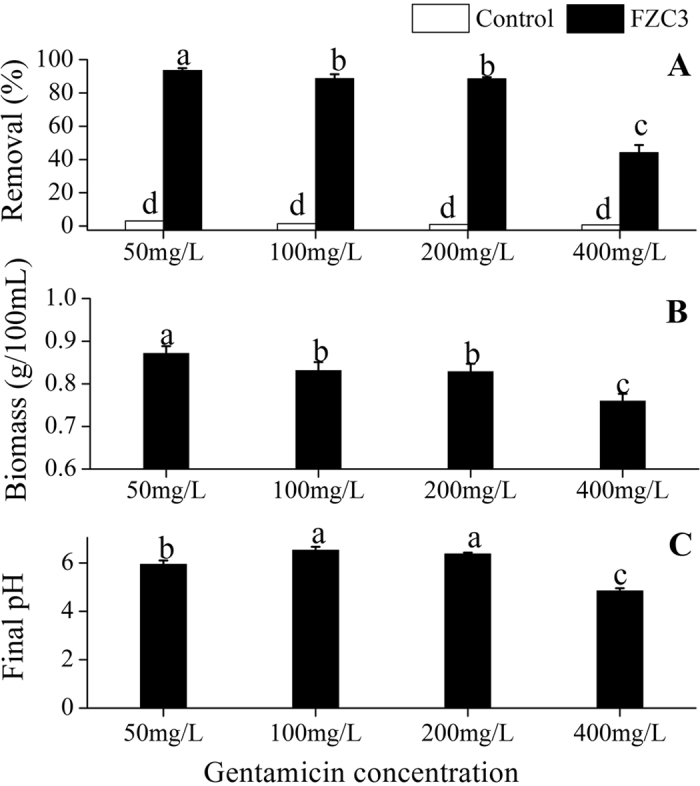
Effects of gentamicin concentration on the gentamicin removal, the biomass of FZC3 and the final pH of the medium. (**A**) Gentamicin removal by day 7 in response to different gentamicin concentrations. (**B**) Biomass of FZC3 in 100 mL of medium by day 7 in response to different gentamicin concentrations. (**C**) Final pH in the medium by day 7 in response to different gentamicin concentrations. The mean values and standard deviations (*error bars*) are presented (n = 3). *Data bars* with the same letter are not significantly different from one another, as determined by Duncan’s test (*p* < 0.05). The figure was created by Origin 8.5.

The initial gentamicin concentration also affected its removal by FZC3. FZC3 removed proportionally more gentamicin (93–88%) at a lower gentamicin concentration (50 mg L^−1^ to 200 mg L^−1^; Fig. 4A). However, gentamicin removal decreased sharply to 44% when 400 mg L^−1^ of gentamicin was added. The amount of fungal biomass produced followed the same trend as gentamicin removal (Fig. 4B), possibly because gentamicin might be toxic to FZC3 at a high concentration, inhibiting its growth. The final pH in the spent medium was also significantly affected by the gentamicin concentration (*p *< 0.05, Fig. 4C). Lin *et al.*^19^ reported that the bioremediation efficiency of cephalexin was not significantly affected by an increase in the antibiotic concentration. In this study, the removal efficiency decreased when the gentamicin concentration was higher. A high concentration of gentamicin had a negative effect on the growth and activity of FZC3.

**Figure 5:**
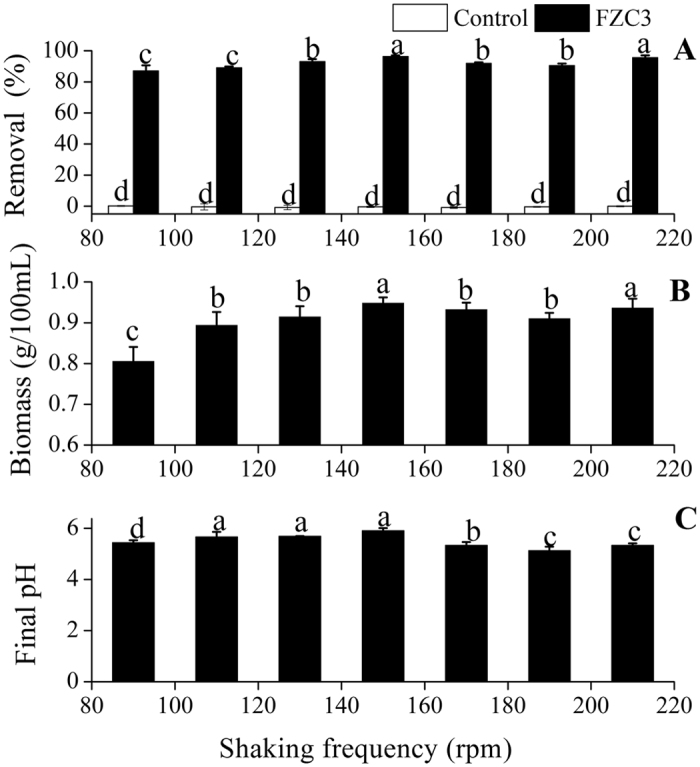
Effects of the shaking frequency on the gentamicin removal, the biomass of FZC3 and the final pH of the medium. (**A**) Gentamicin removal by day 7 in response to different shaking frequencies. (**B**) Biomass of FZC3 in 100 mL of medium by day 7 in response to different shaking frequencies. (**C**) Final pH of the medium by day 7 in response to different shaking frequencies. The mean values and standard deviations (*error bars*) are presented (n = 3). *Data bars* having the same letter are not significantly different from one another by Duncan’s test (*p* < 0.05). The figure was created by Origin 8.5.

FZC3 was grown at constant temperature on an orbital shaker at seven shaking frequencies from 90 to 210 rpm. As the shaking frequency increased from 90 rpm to 150 rpm, gentamicin removal increased from 87.1% to 96.3%, respectively (Fig. 5A). The amount of fungal biomass in 100 mL of medium rose from 0.71 g to 0.95 g (Fig. 5B). Shaking at 210 rpm also gave a high gentamicin removal of 95.6%. The shaking frequency was shown to be an important factor that affected the fungal biomass by changing the contact area between the microorganism and medium and increasing the dissolved oxygen concentration in submerged fermentation^20^,^21^. Certainly, aeration has an influence on complex physiological and biochemical processes. As shown in Fig. 5C, the culture at 150 rpm showed a significantly higher pH than other treatments.

**Figure 6:**
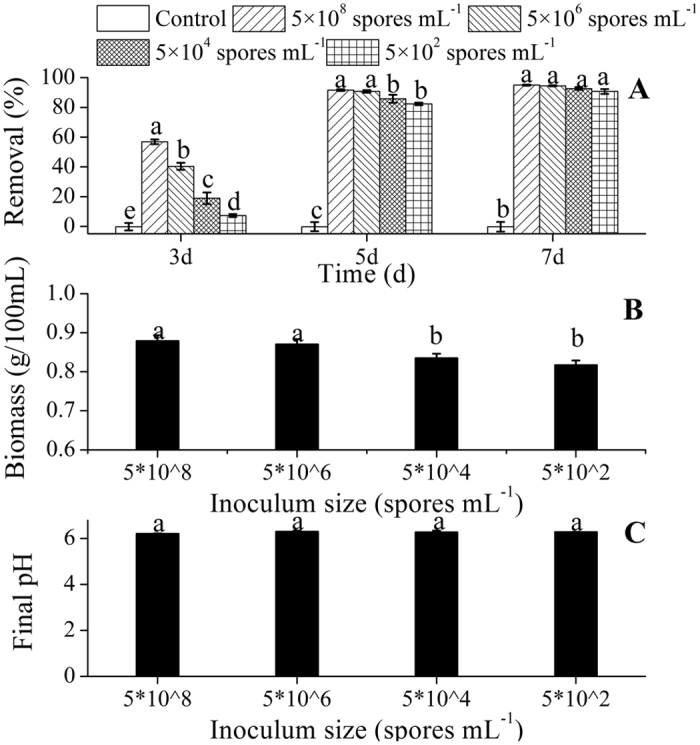
Effects of the inoculum size on the gentamicin removal, the biomass of FZC3 and the final pH of the medium. (**A**) Gentamicin removal over a 7-day period in response to different inoculum sizes. (**B**) Biomass of FZC3 in 100 mL of medium by day 7 in response to different inoculum sizes. (**C**) Final pH of the medium by day 7 in response to different inoculum sizes. The mean values and standard deviations (*error bars*) are presented (n = 3). *Data bars* with the same letter are not significantly different from one another, as determined by Duncan’s test (*p* < 0.05). The figure was created by Origin 8.5.

Gentamicin removal by FZC3 was affected by the inoculum size. Cultures inoculated with 5 × 10^8^, 5 × 10^6^, 5 × 10^4^, and 5 × 10^2^ spores mL^−1^ removed 56.9%, 40.4%, 18.9%, and 7.3%, of the gentamicin, respectively, by the 3^rd^ day (Fig. 6A). Continued cultivation narrowed the differences among the four treatments. A higher number of spores used for inoculation produced a greater fungal biomass (Fig. 6B), and more gentamicin was removed. Thus, the results indicated that inoculum size was a critical factor that affected fungal growth and antibiotic removal, especially in the early days of fermentation. The inoculum size did not significantly affect the final pH of the spent medium (Fig. 6C).

**Figure 7:**
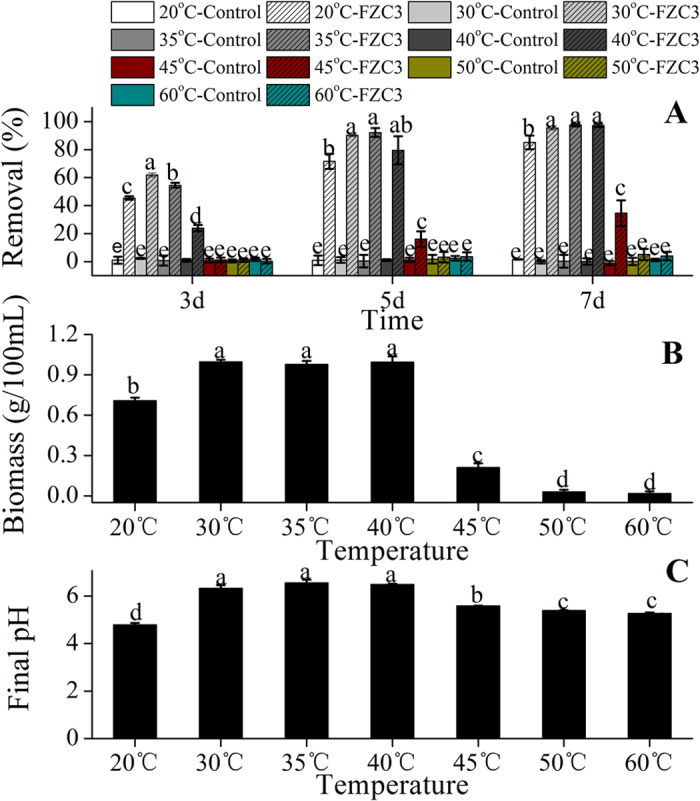
Effects of the temperature on the gentamicin removal, the biomass of FZC3 and the final pH of the medium. (**A**) Gentamicin removal over a seven day period in response to different temperatures. (**B**) Biomass of FZC3 in 100 mL of medium by day 7 in response to different temperatures. (**C**) Final pH of the medium by day 7 in response to different temperatures. The mean values and standard deviations (*error bars*) are presented (n = 3). *Data bars* with the same letter are not significantly different from one another, as determined by Duncan’s test (*p* < 0.05). The figure was created by Origin 8.5.

Temperature is usually regarded as the most important factor that affects the microbial growth of all eco-physiological parameters. We determined the gentamicin removal at temperatures from 20 °C to 60 °C, and the results are summarized in Fig. 7A. The maximum antibiotic removal (61.8%) was observed at 30 °C on day 3. At 35 °C, 92.1% and 97.5% of the antibiotic was removed by day 5 and day 7, respectively. The growth of *Aspergillus terreus* FZC3 was obviously inhibited when the temperature was higher than 45 °C (Fig. 7B), and a lower gentamicin removal was obtained, suggesting that this strain could not be used in a high-temperature fermentation or in the thermophilic phase of composting. Some enzymes produced by *Aspergillus terreus*, such as endoglucanase and cellulose, can be stable up to 60 °C^22^,^23^. Therefore, further studies should focus on the effects of temperature on gentamicin-degrading enzymes. Unsuitably high or low temperatures may lead to acidification of the medium (Fig. 7C).

**Figure 8:**
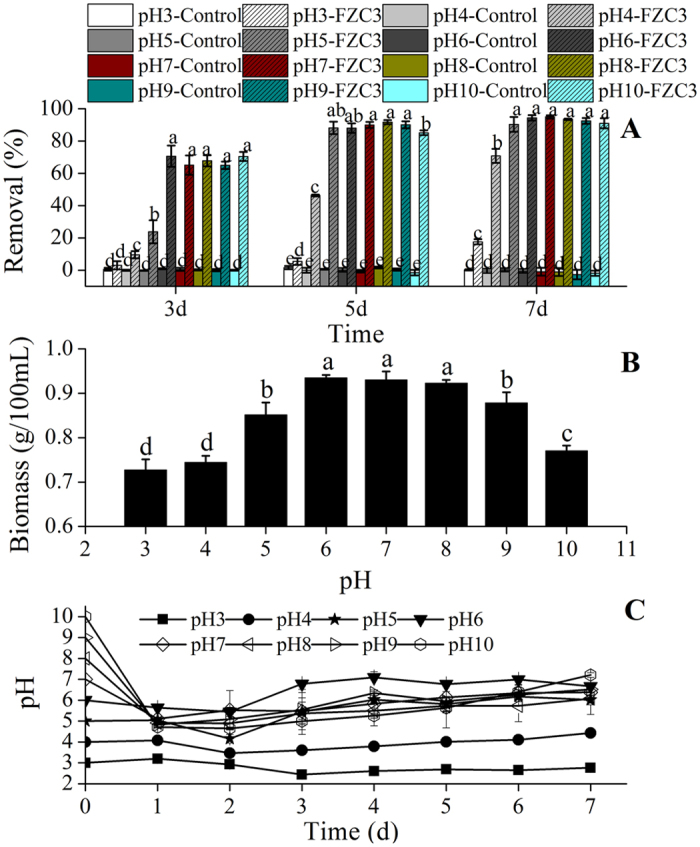
Effects of initial pH of the medium on the gentamicin removal, the biomass of FZC3 and the pHs of the medium. (**A**) Gentamicin removal over a seven day period in response to different initial pHs. (**B**) Biomass of FZC3 in 100 mL of medium by day 7 in response to different initial pHs. (**C**) pH changes of the medium over seven days in response to different initial pHs. The mean values and standard deviations (*error bars*) are presented (n = 3). *Data bars* with the same letter are not significantly different from one another, as determined by Duncan’s test (*p* < 0.05). The figure was created by Origin 8.5.

The initial pH of the growth medium and its dynamics during cultivation are critical parameters that affect both mycelial growth and enzyme production by the fungus. *Aspergillus terreus* FZC3 was able to grow well at a relatively wide pH range from 3.0 to 10.0 (Fig. 8B). It was surprising that >60% and >90% of gentamicin was removed by day 3 and day 7, respectively, when the initial pH of the growth medium changed from 6 to 10 (Fig. 8A). Although FZC3 grew slowly and produced a biomass of 0.87 g and 0.77 g in 100 mL of medium on day 7 at an initial pH of 9.0 and 10.0, respectively, which was less than at pH 6.0 to 8.0 (>0.87 g), it maintained a high gentamicin reduction ability with a reduction rate ranging from 91% to 92%. Compared to these alkaline conditions, an acidic initial pH in the culture broth had adverse effects on fungal growth and antibiotic removal. Although FZC3 reached a similar biomass by day 7 at the two lower pHs, its gentamicin removal ability at an initial pH of 4.0 was much greater than that at an initial pH of 3.0 (Fig. 8C), which suggested that the lower initial pH of the medium and its change during cultivation had a larger effect on the degrading enzymes or adsorption sites than on the amount of fungal biomass produced. This finding also means that the amount of biomass produced was not the only factor that determined gentamicin removal. High gentamicin removal at an initial pH of 5.0 (90.3%) by day 7 suggested that the activity of FZC3 improved as the pH in the culture medium moved closer to a slightly acidic optimal value. This is also apparent in Fig. 8C. In cultures with an initial pH >6, the pH dropped to approximately 5 in the first 24 h. This trend was observed in cultures with an alkaline pH, in which the growth of FZC3 swiftly lowered the medium pH to a final pH of approximately 6.0–6.5. This characteristic of FZC3 could make it widely applicable for the treatment of solid and liquid wastes produced during gentamicin production because the waste is usually alkaline.

### Mode of action of gentamicin removal

**Figure 9:**
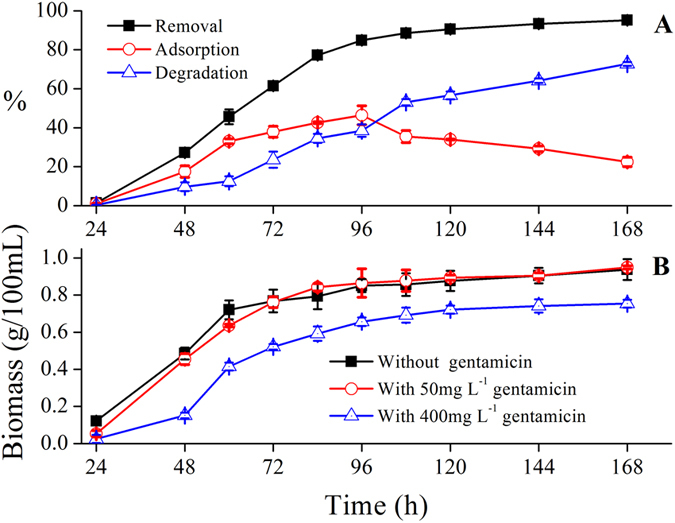
Gentamicin removal and fungal biomass growth over 7 days of fermentation. (**A**) Changes in gentamicin removal, adsorption, and degradation using FZC3 under optimized conditions. (**B**) Growth of FZC3 incubated in 100 mL of LPD at pH 6 with different concentrations of gentamicin (0, 50 and 400 mgL^−1^) at 30 °C and 150 rpm orbital shaking. The inoculum size was 1 × 10^8^ spores mL^−1^. The mean values and standard deviations (*error bars*) are presented (n = 3). The figure was created by Origin 8.5.

Biosorption and biodegradation are bioremediation methods^14^. In this study, the pH and gentamicin concentration in the control did not significantly change after 168 h (data not shown), indicating that gentamicin removal was due to fungal growth and not to interactions with the medium components. The different removal trends of biosorption and biodegradation indicate that gentamicin was reduced by both biosorption and biodegradation (Fig. 9A). Most of the gentamicin (77%) was removed in the first 84 h, which shows a striking similarity with fungal growth in the broth with 50 mg L^−1^ gentamicin (as seen in Fig. 9B). Adsorption and degradation increased until 84 h as the amount of the antibiotic removed increased. Later, the adsorption decreased remarkably and degradation became dominant. The rapid growth of the fungus during the exponential phase provided for a rapid increase of adsorption sites. Degradation could not eliminate the adsorbed antibiotic. Later when growth slowed, biosorption was far slower than degradation by the mature fungal biomass, which resulted in a sharp decrease in adsorption and an increase in biodegradation. Finally, by day 7, the amount of biodegradation was 3.3 times that of biosorption. We can speculate that *Aspergillus terreus* FZC3 took up the antibiotic and subsequently degraded it, similar to the myco-remediation technique described by Migliore *et al.*^8^. Consequently, biodegradation is the final fate of gentamicin in this fermentation system. However, further study of the mechanism is required.

A low antibiotic concentration (50 mg L^−1^) did not have a negative effect on the final weight of FZC3 at hour 168 (approximately 0.93 g in 100 mL of medium) (Fig. 9B). A similar result was observed in an earlier experiment with *Pleurotusostreatus*, in which the fungus in a medium containing 50 mg L^−1^ of oxytetracycline was inhibited at the start and ultimately reached the same final weight as the controls^8^. However, a low antibiotic concentration did exhibit some inhibiting effect during the initial stage of incubation (in the first 72 h). Later, FZC3 adapted to the environment. The amount of biomass produced in a medium containing 400 mg L^−1^ gentamicin was less (approximately 0.75 g in 100 mL of medium) than in the absence of gentamicin or if it was present at a lower concentration. At a higher antibiotic concentration, the fungus also took a longer time to enter the exponential growth phase.

## Materials and Methods

### Chemicals

Gentamicin (purity, 99.99%) was obtained from the Zhichu Pharmaceutical Factory (ZPF; Shandong province, China). A standard stock solution of 10 g L^−1^ gentamicin was prepared with sterile ultrapure water and stored at 4 °C. Lower concentrations of standard solutions were made by diluting the stock solution with sterile ultra-purified water. Methanol and chromatographic grade trifluoroacetic acid (TFA) were purchased from Fisher Scientific (Massachusetts, USA). Ultrapure water was prepared with a Milli-Q Advantage A10 system from Millipore (Massachusetts, USA). All other chemicals that were used were of analytical grade.

### Isolation and screening of the gentamicin-removing fungi

The fungal strains were isolated from various wastes arising from gentamicin production at ZPF (*e.g.,* fermentation wastewater, anaerobic jar sludge, aerobic tank sludge and bio-solids). Martin’s substrate (MS) sterilized at 121 °C for 30 min was placed in sterile Petri dishes and inoculated with approximately 0.5 g of gentamicin production waste samples. The cultures were incubated at 30 °C. After 5 days, the developing fungal thalli were transferred onto potato dextrose agar (PDA) in sterile Petri dishes supplemented with 100 mg L^−1^ gentamicin and were incubated at 30 °C. After another 5 days, the mycelia were scraped into an Erlenmeyer flask containing MSM with 100 mg L^−1^ of gentamicin as the sole carbon source. They were also grown in 1/10 LPD with 100 mg L^−1^ of gentamicin to confirm gentamicin removal by co-metabolism. The two types of media were incubated on a rotary shaker at 30 °C and 150 rpm. The same media without microorganisms were treated under the same conditions as controls. Weekly for four weeks, the gentamicin that was in the media was extracted and quantified by a method described in Chinese Pharmacopoeia (2010)^24^. In this study, all the Erlenmeyer flasks were wrapped in tinfoil to prevent the photo degradation of gentamicin. All the treatments were performed in triplicate.

### Taxonomic identification of FZC3

The isolated fungus strain that showed the best gentamicin removal was designated as FZC3 and was grown on PDA medium for 5 days at 30 °C. High-molecular-weight DNA was obtained using the CTAB method^25^. DNA fragments covering partial 18S rRNA, ITS1, 5.8S rRNA, ITS2, and partial 28S rRNA sequences were amplified and sequenced^11^. The sequences were compared with known sequences in NCBI using the BLAST programs. In addition, FZC3 was also morphologically analyzed^16^. FZC3 was stored under preservation number CGMCC 12072 under the Genebank accession number of KU170490.

### Parameter optimization

Single factor tests were applied to determine the best culture conditions for gentamicin removal by FZC3. The parameters tested included the medium concentration (1/1–1/20), the initial gentamicin concentration (50–400 mg L^−1^), the shaking frequency (90–210 rpm), the inoculum size (5 × 10^2^–5 × 10^8^ spores mL^−1^), the temperature (20–60 °C) and the initial pH (3.0–10.0). The value of the parameter that gave the highest gentamicin removal rate was used for subsequent steps of the study. For example, to determine the effect of the LPD medium concentration on gentamicin removal, 5 × 10^8^ spores mL^−1^ were grown in 250-mL Erlenmeyer flasks containing 100 mL of 1/1, 1/5, 1/10, 1/15, 1/20 diluted medium and 100 mg L^−1^ gentamicin at 30 °C, pH 6, and 150 rpm. The test of initial gentamicin concentration was conducted at the optimized medium concentration, while other parameters were maintained the same as the previous experiment. Other parameters were similarly optimized. In this study, an uninoculated medium was used as the control treatment. Each treatment was performed in triplicate.

### Mode of action of gentamicin removal

To determine whether gentamicin removal by strain FZC3 resulted from biosorption, biodegradation or both, FZC3 was grown in LPD medium under optimal conditions in triplicate, and the LPD medium and the FZC3 thalli were collected at different times ranging from 24 to 168 h. The thalli were separated from the LPD medium by vacuum filtration using a 0.45-μm membrane filter. A 1.5-mL aliquot of the separated liquid was transferred into a 2-mL microcentrifuge tube and centrifuged for 15 min at 13,201 × *g*. The supernatant was then injected into an autosampler vial through a 0.22-μm membrane filter, and the gentamicin concentration in the supernatant was determined by HPLE-ELSD^24^. The amount of gentamicin absorbed by FZC3 was assessed by the following procedure. The separated fungal thalli were treated with 100 mL of 20 mM TFA in a 250-mL Erlenmeyer flask wrapped in tinfoil and shaken on an orbital shaker at 150 rpm. After 2 h, the gentamicin concentration in this desorption solution was determined as described above. The mycelial mass in the media of different gentamicin concentrations (0, 50 and 400 mg L^−1^) was determined using the methods described in a previous study^11^.

The removal (*R*), adsorption (*A*), and degradation (*D*) were calculated using the following equations:


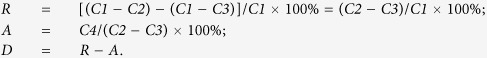


where:

*C1* is the initial concentration of gentamicin in the fermentation broth;

*C2* is the concentration of gentamicin in the control at the end of the experiment;

*C3* is the concentration of gentamicin in the spent medium at the end of the experiment; and

*C4* is the concentration of gentamicin in the desorption solution.

### Statistical analysis

SPSS Statistics ver. 19 software was used for statistical analyses. One-way analysis of variance (ANOVA) was used to determine if treatment means were significantly different, and Duncan’s multiple range test was performed to determine if the individual means were different from each another.

## Additional Information

**How to cite this article**: Liu, Y. *et al.* Gentamicin removal in submerged fermentation using the novel fungal strain *Aspergillus terreus* FZC3. *Sci. Rep.* **6**, 35856; doi: 10.1038/srep35856 (2016).
